# Drug stockouts and treatment delays in African health systems: impact on malaria morbidity and mortality

**DOI:** 10.1097/MS9.0000000000004641

**Published:** 2026-01-06

**Authors:** Emmanuel Ifeanyi Obeagu, Abdulbasit Opeyemi Abdulrahman, John Ibhagbemien Anetor

**Affiliations:** a Department of Biomedical and Laboratory Science, Africa University, Mutare, Zimbabwe; b Department of Medical Laboratory Science, College of Basic Health Sciences, Achievers University Owo, Ondo, Nigeria; c Department of Chemical Pathology and Toxicology, University of Ibadan, Ibadan, Nigeria

**Keywords:** African health systems, drug stockouts, malaria, morbidity and mortality, treatment delays

## Abstract

Malaria remains a leading cause of illness and death in Africa, particularly affecting vulnerable populations such as children and pregnant women. Effective management of malaria relies heavily on timely access to antimalarial medications, primarily artemisinin-based combination therapies. However, drug stockouts and treatment delays are persistent challenges in many African health systems, significantly undermining malaria control efforts and contributing to increased morbidity and mortality. This review explores the multifactorial causes of drug stockouts and treatment delays, including weaknesses in procurement, supply chain management, health infrastructure, and human resources. It highlights how these systemic challenges lead to interruptions in malaria treatment, worsening disease outcomes, and facilitating the spread of drug resistance. The negative impact extends beyond clinical consequences, eroding public trust in healthcare services and pushing patients toward ineffective alternatives. To address these issues, the review underscores the need for comprehensive strategies that strengthen supply chains, improve forecasting and inventory management, and enhance healthcare delivery capacity. Integrating innovative technologies, fostering partnerships, and promoting community engagement are critical to ensuring consistent drug availability and timely treatment. Ultimately, overcoming drug stockouts and treatment delays is essential to reduce the malaria burden and advance toward elimination goals in Africa.

## Introduction

Malaria remains one of the most significant public health challenges in Africa, accounting for the majority of the global malaria burden. Despite substantial progress made over the past two decades, the continent continues to experience high rates of malaria morbidity and mortality, particularly among children under 5 years and pregnant women^[1]^. Effective case management with timely diagnosis and prompt treatment using artemisinin-based combination therapies (ACTs) is a cornerstone of malaria control and elimination strategies recommended by the World Health Organization (WHO). However, achieving these outcomes depends largely on the uninterrupted availability of quality-assured antimalarial drugs at all levels of the health system^[2]^. Drug stockouts, defined as the unavailability of essential medicines at health facilities, are a widespread and persistent problem in many African countries. These stockouts directly affect malaria treatment by causing interruptions or delays in care, forcing patients to seek alternative sources of medication or forgo treatment altogether^[3]^. Treatment delays increase the risk of disease progression from uncomplicated to severe malaria, contributing to higher rates of hospitalization, complications, and death. Moreover, incomplete or inconsistent treatment regimens due to drug shortages can promote the development and spread of drug-resistant parasite strains, undermining malaria control efforts^[4]^.

**HIGHLIGHTS**

- Frequent drug stockouts disrupt timely malaria treatment.
- Treatment delays increase disease severity and death rates.
- Rural areas face the greatest supply challenges.
- Stockout data are often underreported.
- Strengthening supply chains reduces malaria burden.

The complexity of drug stockouts in African health systems stems from multiple, interrelated factors that span procurement, supply chain logistics, health facility management, and broader systemic issues^[5]^. Challenges such as inadequate forecasting of drug needs, delays in procurement processes, poor inventory management, and fragmented supply chains all contribute to the irregular availability of antimalarials^[6]^. In addition, financial constraints, limited infrastructure, and workforce shortages exacerbate these problems. Political instability, corruption, and external shocks like pandemics or natural disasters can further disrupt supply chains and delay drug deliveries^[7]^. Treatment delays are also influenced by systemic health service factors beyond drug availability. Long waiting times, insufficient healthcare staffing, and weak referral systems can prevent timely access to effective malaria treatment, particularly in rural and hard-to-reach areas^[8]^. Delays in diagnosis due to limited access to rapid diagnostic tests (RDTs) and poor community awareness of malaria symptoms further complicate prompt care. These delays can result in avoidable morbidity and mortality, undermining national malaria control programs (NMCP) and increasing the burden on already strained health services^[9]^.

The impact of drug stockouts and treatment delays extends beyond individual patient outcomes, affecting broader public health goals. Consistent and timely treatment is essential not only for reducing morbidity and mortality but also for interrupting malaria transmission cycles within communities^[10]^. Drug shortages can diminish public confidence in health systems, leading to increased reliance on informal providers, self-medication, or traditional remedies, which may be ineffective or harmful. This behavior perpetuates treatment delays and compromises surveillance and control efforts^[11]^. Recognizing the magnitude of this challenge, there has been growing interest in understanding the root causes of drug stockouts and treatment delays and identifying effective strategies to mitigate their impact^[12]^. Interventions such as strengthening supply chain management (SCM), improving health information systems (HIS), investing in human resources, and leveraging technological innovations have shown promise in some settings^[13]^. Moreover, enhancing community engagement and education can empower patients to seek timely care and adhere to treatment regimens, reducing the adverse effects of systemic delays^[14]^. This review aims to provide a comprehensive synthesis of the existing literature on drug stockouts and treatment delays in African health systems and their impact on malaria morbidity and mortality. It seeks to highlight the key challenges and explore potential solutions to improve drug availability and treatment timeliness. Addressing these critical issues is vital to sustain and accelerate progress toward malaria control and eventual elimination on the continent.

## Aim

This review aims to critically examine the prevalence, causes, and consequences of drug stockouts and treatment delays within African health systems, focusing on their impact on malaria morbidity and mortality. It seeks to identify systemic challenges contributing to these issues and explore evidence-based strategies to improve drug availability and ensure timely malaria treatment. Ultimately, the review intends to provide actionable insights to inform policy and strengthen health system responses toward achieving better malaria outcomes in Africa.

## Methods

**Figure 1.**
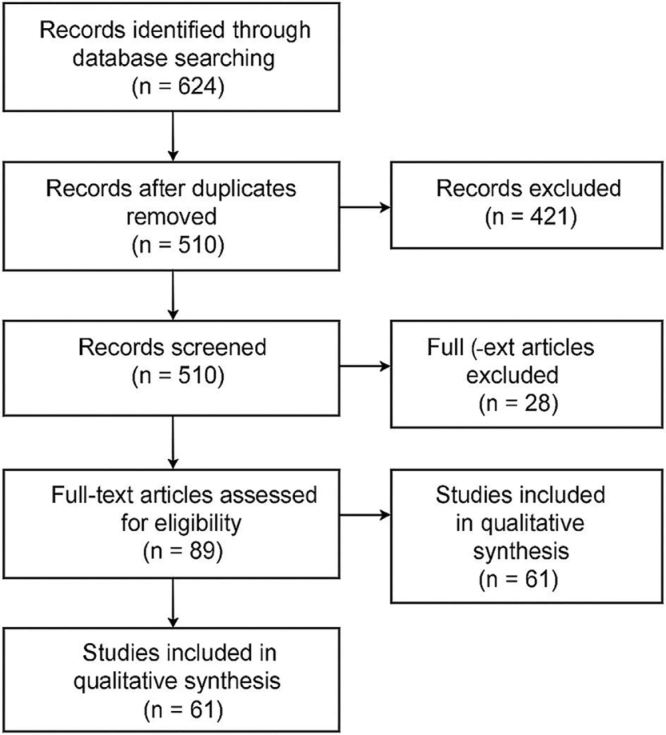
PRISMA flow chart.

This article was conducted as a **Narrative Review** with enhanced methodological transparency to ensure clarity, reproducibility, and coherence, in line with the reviewers’ recommendations (Fig. 1). The aim of the review was to synthesize current evidence on drug stockouts and treatment delays in African health systems and their impact on malaria morbidity and mortality. Although narrative in design, the review incorporated structured elements typically associated with more rigorous review methodologies – such as predefined search strategies, eligibility criteria, and systematic data organization – to strengthen reliability and interpretability.

### Search strategy

A comprehensive literature search was conducted across four major databases – **PubMed, Scopus, Web of Science**, and **African Journals Online** – covering the period from **January 2000 to January 2025**. Additional gray literature was sourced from WHO reports, Global Fund publications, UNICEF supply chain documents, NMCP bulletins, and reference lists of included studies.

### Search terms

The search strategy combined Medical Subject Headings and free-text terms related to drug stockouts, malaria, and African health systems. A representative search string applied in PubMed (and adapted for other databases) was:

**(“drug stockouts” OR “drug shortage” OR “medicine stockout” OR “pharmaceutical shortage”)**

**AND (“malaria” OR “antimalarial” OR “ACTs” OR “artemisinin-based combination therapy”)**

**AND (“Africa” OR “African health systems” OR list of individual African countries).**

All searches were limited to articles published in **English**.

### Eligibility criteria

Given the narrative nature of the review, broad but clearly defined inclusion criteria were applied to ensure comprehensive coverage of relevant evidence.

### Inclusion criteria

- Studies focusing on African countries.
- Articles examining drug stockouts, treatment delays, antimalarial availability, malaria outcomes, or health system-related barriers.
- Peer-reviewed primary studies (quantitative, qualitative, or mixed methods), reviews, policy analyses, national surveillance reports, and technical documents.
- Publications from 2000 to 2025.
- English-language publications.

### Exclusion criteria

- Studies not related to malaria.
- Publications focusing on drug shortages outside Africa.
- Articles without relevance to treatment availability, malaria morbidity, or systemic determinants.
- Non-English publications.

### Study selection process

Two reviewers independently screened the titles and abstracts of all identified records to assess relevance. Full texts of potentially eligible articles were retrieved and evaluated against the inclusion criteria. Discrepancies were resolved through discussion and consensus. Although formal systematic screening tools were not used, efforts were made to ensure consistency and transparency throughout the selection process.

### Data extraction and organization

Data from included sources were extracted manually using a structured template designed to facilitate thematic comparison. Extracted information included:

- Study authors, publication year, and country.
- Study design and setting.
- Type of antimalarial commodity affected (e.g., ACTs, RDTs, and artesunate).
- Reported frequency, duration, or patterns of stockouts.
- Drivers and systemic factors contributing to stockouts.
- Documented or inferred treatment delays.
- Clinical and public health consequences (morbidity, mortality, transmission, and health-seeking behavior).
- Proposed or implemented mitigation strategies.

Extracted data were organized into descriptive tables and analytical summaries to support the narrative synthesis.

### Quality consideration of sources

Although narrative reviews do not traditionally employ formal risk-of-bias tools, a **basic quality consideration** was integrated to strengthen the credibility of findings. Each included study was examined for clarity of objectives, methodological coherence, adequacy of data reporting, and relevance to the review topic. This qualitative appraisal did not exclude studies but informed the interpretation of evidence, particularly when findings were inconsistent or derived from small or context-specific studies.

### Methodological limitations

In line with best practices for narrative reviews, we explicitly acknowledge inherent limitations:

- Possible selection and publication bias.
- Heterogeneity of study designs and reporting standards.
- Absence of formal risk-of-bias tools.
- Limited comparability across countries and regions.
- Inability to establish causality.

### Causes of drug stockouts and treatment delays

Drug stockouts and treatment delays in African health systems arise from a complex interplay of factors across multiple levels of healthcare delivery. One of the primary causes is weaknesses in SCM^[15]^. Inefficient procurement processes, including delays in order placements, bureaucratic hurdles, and lack of timely funding, often result in late or insufficient delivery of antimalarial drugs to health facilities^[16]^. Furthermore, poor forecasting and inaccurate quantification of drug needs can lead to either overstocking or understocking, both of which disrupt consistent drug availability^[17]^. Inadequate infrastructure and logistics further exacerbate the problem. Many rural and remote health facilities face challenges related to transportation, storage, and inventory management^[18]^. Limited cold chain facilities and poor storage conditions can result in drug spoilage or expiration, reducing the usable stock. Additionally, frequent power outages and a lack of proper warehouse management systems contribute to stock degradation and inaccurate inventory tracking, making it difficult to anticipate shortages in advance^[19]^.

Human resource constraints also play a significant role. A shortage of trained health workers and pharmacists can impair the effective management of medicines, including ordering, record-keeping, and stock monitoring^[20]^. This can lead to mismanagement or diversion of drugs, unintentionally causing stockouts. Moreover, inadequate training in supply chain practices means that many facilities lack the capacity to implement efficient inventory control or respond promptly to emerging shortages^[21]^. Financial limitations at both national and facility levels often restrict the ability to maintain a stable supply of antimalarial medications. Budget shortfalls and competing healthcare priorities result in insufficient procurement funding or delayed payments to suppliers^[22]^. This financial instability can disrupt the entire supply chain and lead to gaps in drug availability. Corruption and misallocation of resources further worsen the financial challenges, diverting essential funds away from medicine procurement^[23]^.

Another critical cause of treatment delays relates to health system inefficiencies beyond drug availability. Weak referral systems, limited diagnostic capacity, and overburdened health workers can increase patient waiting times and delay initiation of appropriate treatment^[24]^. In many rural settings, lack of access to RDTs or microscopy prolongs the diagnostic process, which postpones treatment commencement. In addition, geographic barriers such as long distances to health facilities and poor transportation infrastructure contribute to delays in care-seeking^[25]^. Community-level factors also influence treatment timeliness. Low health literacy and cultural beliefs may discourage early healthcare seeking or lead patients to seek traditional remedies first, delaying access to effective antimalarial therapy^[26]^. Economic constraints, including costs of travel and lost income, can further postpone visits to health facilities. These behavioral and social determinants often interact with systemic supply challenges, compounding delays in receiving appropriate malaria treatment^[27]^. External shocks, such as political instability, natural disasters, and pandemics, disrupt supply chains and health service delivery. For instance, the COVID-19 pandemic caused widespread interruptions in global and local medicine supply chains, exacerbating drug shortages in many African countries^[28]^. Political conflicts may restrict the movement of goods and personnel, leading to prolonged stockouts and treatment delays. These unpredictable events underscore the vulnerability of health systems and the urgent need for resilient supply chain mechanisms (Table 1)^[29]^.

**Table 1:** Causes of drug stockouts and treatment delays in African health systems

Category	Specific causes	Description/examples
1. Supply chain inefficiencies	Poor quantification and forecasting	Inaccurate estimation of ACT and diagnostic needs leading to under-procurement.
	Delayed procurement cycles	Long tender processes, bureaucratic approvals, supplier delays.
	Weak warehousing and storage systems	Inadequate stock management systems and cold-chain weakness for certain commodities.
	Transportation and distribution challenges	Poor road networks, fuel shortages, and limited last-mile delivery.
2. Health system constraints	Inadequate human resources	Insufficient pharmacy staff, poor stock reporting, and limited training in supply management.
	High patient volumes	Demand surges during malaria peaks, overwhelming facility supplies.
	Fragmented logistics management	Lack of integrated national digital logistics systems.
3. Financial and economic barriers	Funding gaps in national malaria programs	Insufficient domestic budgets, reliance on donor funding cycles.
	Delays in the disbursement of funds	Interruptions affecting the timely procurement of ACTs and RDTs.
	Currency fluctuations and import costs	Increased cost of importing antimalarials and medical supplies.
4. Governance and policy issues	Weak accountability mechanisms	Limited oversight of commodity distribution and stock tracking.
	Leakage, pilferage, and corruption	Diversion of antimalarials into private markets, inflating supply chain losses.
	Poor policy coordination	Misalignment between national malaria programs, central medical stores, and facility-level systems.
5. External and environmental factors	Seasonal variations	Stockouts during high-transmission rainy seasons due to inadequate prepositioning.
	Epidemics and public health emergencies	Resources diverted during outbreaks (e.g., Ebola and COVID-19).
	Political instability and conflict	Disruptions in supply routes and facility access.
6. Market and industry challenges	Global ACT supply shortages	Delays in international production or export restrictions.
	Limited local pharmaceutical manufacturing	Dependence on imported antimalarials.
	Supplier unreliability	Delivery delays or quality issues leading to rejected consignments.
7. Facility-level operational issues	Incomplete or inaccurate stock reporting	Reporting gaps that prevent timely replenishment.
	Poor inventory management	Failure to monitor minimum stock levels or reorder points.
	Lack of buffer or emergency stock	Facilities unable to cushion against sudden demand spikes.

### Impact on malaria morbidity and mortality

Drug stockouts and treatment delays have profound consequences on malaria morbidity and mortality across African health systems. When essential antimalarial medications are unavailable, patients are either forced to delay seeking treatment or resort to incomplete or ineffective therapies, increasing the likelihood of disease progression^[30]^. Delays in treatment can allow uncomplicated malaria to escalate into severe forms, characterized by complications such as cerebral malaria, anemia, and multiorgan failure, which are often fatal if not promptly managed^[31]^. The lack of timely and effective treatment directly contributes to higher hospitalization rates and mortality, especially among high-risk groups such as young children and pregnant women^[32]^. Studies have consistently shown that interruptions in the availability of ACTs, the frontline treatment for uncomplicated malaria, are associated with increased case fatality rates. In regions experiencing frequent stockouts, the burden of severe malaria and deaths is disproportionately higher, undermining national and global efforts to reduce malaria-related mortality^[33]^.

Furthermore, inconsistent access to antimalarial drugs facilitates the emergence and spread of drug-resistant *Plasmodium* strains. Incomplete or suboptimal treatment due to stockouts promotes parasite survival and selection pressure, which accelerates resistance development^[34]^. Drug resistance not only jeopardizes individual patient outcomes but also threatens the efficacy of current treatment protocols, potentially leading to larger outbreaks and increased malaria transmission^[35]^. Beyond direct clinical outcomes, drug stockouts and treatment delays erode public confidence in the healthcare system, reducing health-seeking behavior and adherence to treatment regimens. Patients may turn to informal providers or traditional medicines, which are often ineffective, further delaying appropriate care^[36]^. This cycle perpetuates the disease burden, hampers accurate malaria surveillance, and challenges control programs in effectively targeting interventions.

Health systems facing frequent drug shortages also experience increased economic burdens due to prolonged illness, repeat healthcare visits, and higher treatment costs associated with managing severe malaria cases. These economic consequences affect both health services and households, particularly in resource-constrained settings where out-of-pocket expenses can be catastrophic^[37]^. Moreover, the cumulative effect of stockouts and treatment delays jeopardizes the progress made under global initiatives such as the WHO Global Technical Strategy for Malaria, which aims to reduce malaria morbidity and mortality by at least 90% by 2030. Without addressing these systemic challenges, the risk of malaria resurgence remains high, threatening the goal of malaria elimination in Africa (Table 2)^[38]^.

**Table 2:** Impact on malaria morbidity and mortality

Impact domain	Description of effect	Evidence summary from African settings
Increased uncomplicated malaria cases	Drug stockouts prevent timely access to first-line antimalarial therapy, leading to rising outpatient malaria cases and prolonged symptom duration.	Studies from Tanzania, Uganda, and Nigeria consistently link ACT stockouts with spikes in confirmed malaria cases, especially during high-transmission seasons.
Progression to severe malaria	Delayed treatment (>24–48 hours) allows parasitemia to rise, increasing the risk of severe anemia, cerebral malaria, and multiorgan involvement.	Up to 40% of severe pediatric malaria admissions in some settings occur following prior inability to access ACTs due to stockouts or facility-level delays.
Increased hospital admissions	Patients who cannot access timely treatment often deteriorate clinically and require inpatient care.	Hospitals in Malawi and Ghana report admission surges of 20–60% during prolonged stockouts of artesunate and ACTs.
Higher case fatality rates (CFRs)	Treatment delays increase parasite biomass and reduce the effectiveness of rescue therapy, especially in young children.	CFRs for severe malaria rise 2–4 fold during documented stockout periods in multiple sub-Saharan African studies.
Reemergence of malaria outbreaks	Lack of effective first-line treatment increases transmission reservoirs, fueling local outbreaks.	Outbreak investigations in Kenya and South Sudan have traced epidemic amplification to sustained ACT stockouts at community and primary-care levels.
Increased use of ineffective or substandard alternatives	Patients turn to older or non-recommended drugs (e.g., chloroquine, sulfadoxine–pyrimethamine), leading to treatment failure.	Reports from DRC, Nigeria, and Mozambique show high treatment failure rates when patients use informal-market drugs during stockout periods.
Delayed case management in pregnant women	Treatment delays in pregnancy increase risks of placental malaria, severe anemia, stillbirth, and low birth weight.	Maternal health studies in Burkina Faso and Tanzania highlight a significant rise in obstetric complications when IPTp or ACTs are unavailable.
Economic burden on households	Disease progression from uncomplicated to severe malaria increases costs for admission, diagnostics, and transport.	Household expenditure analyses show out-of-pocket costs doubling or tripling during stockout periods, worsening catastrophic health spending.
Community-level mortality surges	When stockouts coincide with high-transmission periods, community-level mortality may rise due to delayed access to effective treatment.	Cluster surveillance in Sierra Leone and northern Uganda links temporal mortality peaks to periods of medicine unavailability, especially for children <5 years.

### Health system challenges

The persistent issue of drug stockouts and treatment delays in malaria management reflects broader health system challenges that many African countries face. One major challenge is the fragmented and under-resourced supply chain infrastructure^[39]^. Many countries lack integrated, real-time inventory management systems, making it difficult to track stock levels accurately and respond promptly to shortages. This fragmentation often results from multiple parallel supply chains managed by different stakeholders, including governments, donors, and nongovernmental organizations, which complicates coordination and accountability^[40]^. Another critical challenge is inadequate human resources capacity within the health sector. There is a shortage of trained personnel skilled in pharmaceutical logistics, SCM, and data reporting^[41]^. This workforce gap leads to inefficiencies in ordering, storing, and distributing antimalarial drugs, as well as delays in recognizing and addressing stockouts. Additionally, high staff turnover and insufficient continuous professional development opportunities weaken the institutional memory and capacity to manage complex supply chains effectively^[42]^.

Financial constraints severely limit the ability of health systems to maintain consistent drug availability. Many African countries rely heavily on donor funding for procuring antimalarial medications, making them vulnerable to external funding fluctuations^[43]^. Budgetary constraints at national and facility levels result in delayed or insufficient procurement, payment backlogs to suppliers, and limited investment in supply chain infrastructure. In some cases, corruption and mismanagement further divert scarce resources away from essential drug procurement and distribution activities^[44]^. HIS also present significant challenges. Weak data collection, reporting, and analysis hamper evidence-based decision-making regarding drug procurement and distribution^[45]^. Many health facilities still rely on paper-based records or outdated digital systems that do not communicate efficiently with national databases. As a result, forecasting and quantification of drug needs are often inaccurate, leading to mismatches between supply and demand and contributing to stockouts^[46]^.

Geographical and infrastructural barriers complicate the distribution of antimalarial drugs, particularly in rural and remote areas where malaria burden is often highest. Poor road networks, limited transportation options, and inadequate storage facilities impede the timely delivery of drugs^[47]^. Seasonal factors such as heavy rains can further disrupt supply routes, increasing the risk of stockouts and treatment delays in vulnerable communities^[47]^. Governance and policy challenges also impact drug availability and treatment timeliness. Weak regulatory frameworks, lack of clear policies on SCM, and insufficient oversight mechanisms reduce accountability and transparency^[48]^. Coordination between different levels of government and health sector actors is often suboptimal, leading to duplication of efforts or gaps in service delivery. Moreover, policy inertia and slow adoption of innovations hinder progress in addressing stockout issues effectively^[49]^. External shocks such as political instability, conflicts, and public health emergencies like the COVID-19 pandemic exacerbate existing health system weaknesses. These events disrupt supply chains, divert resources, and overwhelm health facilities, further increasing the risk of antimalarial drug shortages and treatment delays^[50]^. Building resilient health systems capable of withstanding such shocks is essential to ensure continuous malaria care.

### Strategies to improve drug availability and reduce treatment delays

Addressing drug stockouts and treatment delays in malaria care requires a multifaceted approach targeting the root causes within African health systems. Strengthening SCM is fundamental. Implementing integrated, digital inventory management systems can enhance real-time tracking of drug stocks and improve forecasting accuracy^[51]^. Technologies such as mobile-based reporting and electronic logistics management information systems have shown promise in enabling timely identification of shortages and facilitating rapid resupply^[52]^. Capacity building of healthcare workers and supply chain personnel is equally vital. Training programs focused on pharmaceutical logistics, inventory management, and data use can empower staff to effectively manage drug stocks and reduce wastage^[53]^. Establishing dedicated cadres for SCM within the health workforce can ensure sustained expertise and accountability. Continuous professional development and supportive supervision are necessary to maintain high standards^[54]^.

Financial sustainability must be prioritized to ensure uninterrupted drug procurement. Diversifying funding sources, including increased domestic budget allocation and innovative financing mechanisms, can reduce overreliance on external donors^[55]^. Transparent financial management and anti-corruption measures will safeguard resources intended for malaria medicines. Strengthening procurement policies and adopting pooled procurement strategies can improve cost efficiency and timely availability of antimalarials^[56]^. Improving infrastructure and logistics is another critical strategy. Investment in transportation networks, cold chain facilities, and storage capacities at regional and facility levels can minimize drug losses and delivery delays. Leveraging public–private partnerships and community-based distribution networks can extend drug availability to hard-to-reach areas. Additionally, integrating malaria drug supply with other essential health commodities may optimize distribution efficiency^[57]^.

Enhancing HIS to support decision-making is essential. Upgrading from paper-based to digital reporting and establishing interoperable platforms allows for timely data sharing across levels of the health system. Regular data quality audits and feedback mechanisms encourage data-driven supply management. Accurate, timely data facilitate better quantification, reduce stockouts, and improve overall treatment access^[58]^. Community engagement and health education initiatives can reduce treatment delays by promoting early care-seeking behavior and adherence to prescribed antimalarial regimens. Awareness campaigns highlighting the importance of timely treatment and dispelling myths about malaria care empower communities to utilize formal health services promptly. Strengthening collaboration between formal health providers and community health workers ensures that patients receive prompt diagnosis and treatment even in remote settings^[59]^. Policy reforms and governance improvements are necessary to sustain progress. Establishing clear national guidelines on SCM, enforcing regulatory oversight, and fostering coordination among stakeholders enhances accountability and efficiency^[60]^. Integrating malaria control efforts with broader health system strengthening initiatives ensures a holistic approach to addressing drug availability and treatment challenges. Building resilient health systems capable of adapting to external shocks like pandemics or conflicts will further safeguard continuous malaria care (Table 3)^[61,62]^.

**Table 3:** Strategies to improve drug availability and reduce treatment delays

Strategy category	Description of intervention	Expected outcomes	Supporting evidence from African health systems
Strengthening supply chain management	Implement real-time stock monitoring systems, optimize quantification, and reduce procurement bottlenecks.	Fewer stockouts, timely replenishment, improved forecasting accuracy.	Rwanda and Zambia reported substantial reductions in ACT stockouts after adopting electronic LMIS and data-driven procurement.
Decentralized drug distribution models	Shift selected stock management functions to districts or facilities to reduce reliance on central warehouses.	Faster resupply cycles, increased facility autonomy, and reduced administrative delays.	Kenya and Ethiopia show improved continuous ACT availability through district-led distribution systems.
Expansion of community health worker (CHW) programs	Equip CHWs with ACTs, RDTs, and referral tools to enable community-level case management.	Earlier treatment initiation, reduced facility burden, and fewer severe malaria cases.	iCCM programs in Uganda, Malawi, and Burkina Faso significantly reduced treatment delays and child mortality.
Performance-based financing (PBF)	Link facility funding to indicators such as stock availability, accurate reporting, and timely ordering.	Improved accountability, stronger stock management practices, and better service readiness.	PBF schemes in Rwanda and Cameroon demonstrate higher drug availability and improved stock record completeness.
Strengthening local manufacturing capacity	Support regional production of ACTs and essential medicines to reduce reliance on imports and long procurement cycles.	Shorter supply chains, reduced international shipping delays, and improved affordability.	South Africa, Nigeria, and Kenya have shown improved availability of selected medicines through local production initiatives.
Emergency buffer stocks and rapid response systems	Establish national and district-level reserve stocks and rapid redistribution protocols during shortages.	Mitigation of unexpected stockouts, improved resilience during surges or supply disruptions.	Ghana and Tanzania successfully used buffer stocks to maintain ACT availability during global supply interruptions.
Improving health facility logistics and storage	Strengthen inventory management, storage conditions, and staff training in logistics.	Reduced wastage and expiries, more reliable stock records, improved readiness.	Facility-level interventions in Malawi and Mozambique improved stock reliability by enhancing SOP adherence.
Digital health innovations	Use mobile reporting tools, automated alert systems, and supply chain dashboards to track stock levels.	Faster problem identification, real-time redistribution, and early warning of impending stockouts.	SMS- and app-based systems in Tanzania, Uganda, and Nigeria have cut reporting delays and reduced ACT stockouts.
Policy and regulatory reforms	Streamline procurement regulations, reduce customs delays, and harmonize drug registration across regions.	Faster access to quality-assured medicines, fewer administrative obstructions.	Regional harmonization through the East African Community Medicines Regulatory Harmonization (EAC-MRH) program has shortened approval timelines.
Community engagement and accountability mechanisms	Involve communities in monitoring stock levels and reporting shortages through local committees or digital platforms.	Increased transparency, strengthened local oversight, and faster corrective actions.	Community scorecards in Malawi and Zambia improved service responsiveness and reduced prolonged shortages.

## Conclusion

Drug stockouts and treatment delays remain significant barriers to effective malaria control and elimination efforts in Africa, contributing to increased morbidity, mortality, and the potential spread of drug resistance. These challenges stem from multifactorial health system weaknesses, including supply chain inefficiencies, limited human resource capacity, inadequate financing, and infrastructural constraints. Addressing these systemic issues is critical to ensuring timely access to effective antimalarial treatment, especially in rural and underserved communities where the malaria burden is highest. Strengthening supply chains through digital innovations, capacity building, and improved logistics can greatly reduce stockouts and treatment delays. Equally important is securing sustainable financing, enhancing HIS, and fostering community engagement to promote early diagnosis and adherence to treatment. Effective policy reforms and governance are needed to coordinate these efforts and build resilient health systems capable of withstanding external shocks such as pandemics and political instability.
